# The Views of the Working Classes: Their Demand for Hospital Benefit Not as a Matter of Charity, but of Right

**Published:** 1912-12-28

**Authors:** 


### THE VOLUNTARY HOSPITALS AND THE INSURANCE ACT.

### The Views of the Working Classes: their Demand for Hospital Benefit not as a Matter of Charity, but of Right.

OUR GENERAL AND SPECIAL HOSPITALS.

### The British Hospital's Association's Meeting To-day.

A PRACTICAL POLICY.

The Chancellor of the Exchequer has now made it clear that insured persons will need as much as ever the aid of voluntary hospitals in order to obtain the treatment that is given in the in-patient departments, and to a substantial extent that given in the out-patient departments, when special medical or surgical aid is required. In the Government's view the voluntary hospitals would only be carrying out their proper duty in continuing to give this treatment as charity. It is further urged that the subscriptions, donations, and legacies on which these hospitals have depended will be needed as much as ever. In taking this view it seems to us that the Government have clearly not understood all that is involved by setting up a national system of medical benefit under the Insurance Act. They seem to be unacquainted with the fact that for years, and especially during recent years, every voluntary hospital of importance has employed an almoner or almoners with the view to secure that none but those whose circumstances entitle them to medical relief as charity should receive free treatment, as in- or, indeed, as out-patients, except in the casualty department.

The Present Position.

The working classes in all the great centres of industry have more and more spontaneously organised artisans' committees, which have been charged with the twofold duty of raising money for the voluntary hospitals and providing for the admission of the workers to in-patient and other hospital benefit as and when it has been required. Further, the voluntary hospitals, especially in Ireland, have admitted the principle of payment by patients who, like the working classes in industrial centres, have long wished for the opportunity to secure for themselves in-patient treatment, not as a matter of charity but of right. This feeling is one which the introduction of national insurance into this country should stimulate greatly, and, with the wise co-operation of the managers of voluntary hospitals, it should be cultivated until it does in fact bear rare and refreshing fruit. The seed is sown and the principle is admitted through the voluntary hospital system among the general hospitals, where in London 2.6 per cent. of the ordinary income comes from patients' payments, in the provinces and Wales 2.5 per cent., in Scotland 1.6 per cent., and in Ireland 8 per cent. In the special hospitals the principle has taken deeper and firmer root, especially in the out-patient departments. Thus in 1910 in London these special hospitals derived from patients' payments 11.9 per cent. of their ordinary income, in the provincial and Welsh hospitals 16.7 per cent., in Scotland nothing, probably because such contributions are included under work-people's contributions, which yielded 15.8 per cent., and in Ireland 21.9 per cent.

The Government's Grievous Misapprehension.

The Government's intimation that it was not intended to provide the kind of services which can only be obtained in hospitals, and that such treatment is not covered under the National Insurance Act, makes insured persons, from the Government's point of view at any rate, just as necessitous in this matter as they were before. Hospital managers outside London, especially in England, Wales, Scotland, and the north of Ireland, will, we hope, set the Government right upon this point, for they are at present labouring under a grievous misapprehension. It is not possible to give accurately precise evidence through actual figures of the desire which animates masses of hospital patients of the better type to have as a right hospital treatment by payment when they need it at the hospitals. This they cannot, save as charity, as the Chancellor of the Exchequer has pointed out, otherwise obtain, because treatment of this kind is not provided under the Insurance Act. Ever since 1877 there has been an ever-increasing desire, which has periodically found expression in the press, for the opportunity to obtain on payment hospital treatment in pay wards. The better type of patients at voluntary hospitals at present, apart from the working classes, have never been able to organise themselves so as to make their desires fruitful in practice. The present crisis in regard to the voluntary hospitals all over the country, caused by the Insurance Act, should speedily and properly cause these desires to take practical shape, to the greatly added usefulness of the hospitals, with an increase in their economical efficiency, and as centres of education for the training of nurses and medical students.

"Not as a Matter of Charity, but of Right."

The working classes throughout the country have not only desired but, by an exhibition of splendid thrift and manly independence, have obtained, prior to the passing of the Insurance Act, treatment at the voluntary hospitals not as a matter of charity, but of right. This is a point which seems to have escaped the knowledge of the Government. Had the Chancellor of the Exchequer had the point before him, we have little doubt he would not have confined his invitation to the conference on the 17th inst. to the chairmen of London hospitals with medical schools, but would have extended it to representatives of the great provincial, Welsh, Scottish, and North Irish hospitals where the working classes have freed themselves from the taint of charity by the voluntary levies which they have made each year, on a definite basis, to enable their committees to arrange with the voluntary hospital authorities for the reception of those members of their class, who in the course of each year require and must have adequate hospital treatment. We have not space to-day to go into this question in all its details, nor is this necessary for our present object. That object is to bring out clearly the difference which exists between the financial basis on which many of the greater London hospitals rest compared with that of the principal voluntary hospitals outside London, *i.e.*, in the countries we have just enumerated. We are old enough to remember the time when workmen's contributions to hospitals, speaking generally, were practically unknown, with the exception of subscriptions here and there from some works to a hospital, and the organised collections at Glasgow for the Royal Infirmary, and a further and even more remarkable organisation in the Potteries on behalf of the North Staffordshire Infirmary. The last two had been progressively working for twenty years prior to 1870. In 1869 the late Mr. Sampson Gamgee, F.R.S.Edin., organised a fund among the working men of Birmingham to defray the cost of an additional wing and new out-patient department to the Queen's Hospital. On the completion of this work the artisans, to their great credit, desired to continue the organisation and to widen its basis by dividing their contributions amongst the hospitals in that city. A public meeting was held to promote in 1873, a Hospital Saturday collection throughout the industrial establishments in Birmingham for the free benefit of its medical charities. In ten years £45,000 was contributed through Hospital Saturday at a cost of less than 8 per cent. The sum now raised there exceeds £20,000 a year. At that time what Birmingham did all industrial England was wishful to do, and the working-class movement in favour of the voluntary hospitals initiated by the late Sampson Gamgee of blessed memory has been, indirectly, the means of causing the work-people of the greater industrial centres throughout the Kingdom, by their spontaneous efforts to earn hospital treatment not as a matter of charity but of right. In proof of this we need only give a few out of several instances of such movements in the United Kingdom. In 1910 at Stoke-on-Trent the workpeople contributed 63 per cent. of the total ordinary income of the North Staffordshire Infirmary; at Sunderland (Royal Infirmary), 61 per cent.; at Newcastle-on-Tyne (Royal Victoria Infirmary), 56 per cent.; at Leicester (Royal Infirmary), 44 per cent.; in Wales (Swansea General Hospital), 53 per cent.; at Cardiff (King Edward VII.'s Hospital), 32 per cent.; at Glasgow (Western Infirmary), 30 per cent. and (Royal Infirmary) 26 per cent.; at Edinburgh (Royal Infirmary), 23 per cent.; in Ireland (Belfast Royal Victoria Hospital), 29 per cent.

### The Position in London.

Of course, there have been splendid efforts at thrift and self-help on the part of the working classes in the Metropolis, which cannot be regarded, however, in any real sense, as an industrial centre from the artisans' point of view, so that the total contributions from work-people there in 1910, including Hospital Saturday, so far as the Metropolitan general hospitals are concerned, only amounted to 1.7 per cent. of their total ordinary income. This fact proves to demonstration the enormous differences which must underlie the relations to the Insurance Act, its incidence and consequences, in the case of the general hospitals of London or most of them, and of those situated in England, Wales, Scotland, and Belfast. Nearly everywhere except in London, to an increasing extent year by year, the artisans have raised the amount of their contributions to a total sum, and in some cases have exceeded it, which is greater than the whole cost of the hospital treatment they have received at the voluntary hospitals in the centres where these spontaneous levies by the workmen have been splendidly organised, administered, and maintained.

### A Modified System.

We hope that in due course and without much further delay it will be found possible, in the case of many of the larger voluntary hospitals referred to, to enter into an arrangement between the workmen's committees and themselves to credit to a separate fund all the workmen's contributions paid in to the hospital account, and to charge against this fund the actual *pro rata* cost to the hospital of the treatment given to the men sent in from the contributing works. By some such means as this it should speedily be made impossible for any politicians of either party longer to labour under the misapprehension that the best of the working classes throughout this country, prior to the passing of the Insurance Act, have been dependent upon charity and the acceptance of charity during serious illness which has necessitated their admission to treatment at a voluntary hospital. The splendid efforts of thrift and independence, which the figures we have quoted prove, entitle our working men, as insured persons, to stand firmly by their claim to hospital treatment as a right, and not as a matter of charity.

### The "Hospital Saturday Fund Journal's" Declaration.

In this connection we welcome the following strong and just declaration from the authorities of the Metropolitan Hospital Saturday Fund, which appears in No. 80 of the *Hospital Saturday Fund Journal*, dated December 31, 1912:—" As an association supported mainly by insured people for the support of voluntary hospitals it is our duty to urge approved societies on the one hand, and the managers of voluntary hospitals on the other, to see that hospital benefit is provided and paid for. We are of opinion that insured persons, not as a matter of charity but of right, should be entitled to consultative advice and special medical and surgical treatment at hospitals, which, however, should be paid *pro rata* for the advice, treatment, and accommodation provided. But it should be done in such a way as not to impair the voluntary hospital system." We are in entire agreement with this manly declaration of right, and we believe that it will be found accurately to express the sentiments of the leaders of all the great artisan funds in support of the hospitals, especially in the principal centres of industry which we have just enumerated. Whatever the Insurance Act may do it cannot be permitted to interfere with the free levies for the voluntary hospitals by the working classes, or their claim as a right to be entitled to consultative advice and special medical and surgical treatment at the hospitals which they support so splendidly and so gratefully.

### A Representative Workman's Views.

A gentleman of influence and position, who is deservedly popular with the working classes, has been good enough to send us his notes of a conversation with a working man on the Insurance Act. This workman was of the foreman type, thoroughly representative, intelligent, and a fine specimen of the British artisan class. We give the conversation because it is most important that the inside view of the working classes should be generally known and understood. They have shown their value and opinion of the voluntary hospital system by the noble contributions they are increasingly making to the very hospitals which they habitually frequent when ill—a splendid testimony to the high efficiency of the administration of these institutions. All thoughtful persons who have practical knowledge of the social requirements of the nation are, we believe, in favour of an adequate, well-administered, splendidly-organised, and efficient system of national insurance. It must necessarily take a few years in this country as it has taken many years in Germany to arrive at this stage of efficiency. We believe points of view like those which follow if expressed by the working classes would be well calculated to hasten the time when efficiency will mark the whole administration and working of National Insurance in this country. Our working-man friend began:—

### "WHAT I FEEL AND THINK."

The thing won't do. Medical benefit sounds all right, but the term is misleading. The question is, how is it going to be given and what sort is it going to be? The doctor won't be able to attend cases in their own homes; he can't do it. The working man can't go and sit in the doctor's waiting-room amongst a crowd of other patients to wait his turn; he can't afford the time. So it will mean that your 35*s.* to 40*s.* a week man will decide to pay twice. He will pay the insurance and have his own doctor.

What sort of doctoring are we likely to get under the Act? As a general rule it will be the unsuccessful man or the young beginner who will offer himself for the work, and things will be worse as time goes on. An inferior kind of man will be attracted to the profession, the sort of man who will be content with the small salary that he can earn as an Insurance doctor. Now a young man wants to get on, and he knows he must do it by his own hard work; he goes to a place and waits and works up his practice, keeping himself up to date and on the lookout for good work. A young man who starts as a salaried official will be a different sort of man altogether.

Moreover, the Act is bound to hurt the hospitals. People who have to pay insurance, whether working people or others, won't subscribe to the hospitals as they have done in the past. Anything which hurts the hospitals will in the long run damage the medical profession. The hospitals are the places where the doctors learn their work. A man won't learn on an insurance round what he will learn by sticking to his hospital. A friend of mine has been working at a London hospital lately. He had never been there before and I have never been there. He says that they have everything which can possibly be wanted, every new appliance that science has discovered, and all is at the service of the poor patient, and the doctor has experience in the use of all that medical science needs. So it will be a bad day for the working-man patient if the hospitals are damaged in any way.

Then as to the drugs. What sort of medicine are we going to get under the Act? Many medicines are made up with expensive drugs, and if the money provided is not enough for the purchase of these drugs, then we shall get only just something else which "will do."

Then there is another point. What about the man out of work? A working man asks the question to-day: I am out of work now, so my card is not being stamped by an employer. Can I claim benefit when the Act comes into force? The answer is, you will get benefit when you have made twenty-six payments, so that means that a man must fill up his card himself, and how can he do it?

Our correspondent adds: "My friend's conclusion was that it was very misleading to talk about medical benefit, and it wants thinking about as to what it really means."

### The General and Special Hospitals of London.

In addition to what we have already stated, it must be remembered that the hospitals of London do in great measure confer hospital benefits on a considerable number of patients who are residents outside the metropolitan area. For very many years they were regarded and treated as places to which difficult or doubtful cases could be sent for consultation with the most eminent members of the profession, and where they would, in cases of difficulty, be correctly diagnosed and adequately treated. As the means of communication have been increased and the population has grown, this view has necessarily become modified, and to-day many of our great cities have voluntary hospitals of the highest importance and greatest efficiency, which are quite capable of adequately treating all cases of every kind needing hospital care. The London hospitals, too, have on the whole much larger endowments than most other voluntary hospitals outside the Metropolis. They further possess the invaluable financial strength which the King's Hospital Fund has brought them, and they have in addition the continued vitality and ever-extending influence which a hospital gains from its association with a great medical school. On these grounds, and for other reasons which we might advance, the general and special hospitals in London are undoubtedly more favourably placed, whilst their circumstances differ greatly from those in the provinces, Scotland, and Ireland. Several of these reasons were brought out in the statement of Mr. E. W. Morris, the secretary of the London Hospital, which was published in the *Daily Telegraph* of the 18th instant, which everyone interested should make a point of reading. Mr. Morris states "The Act promised to give 'adequate medical relief,' and Mr. Lloyd George, in describing the operation of the Act, constantly referred to 'no taint of charity.' When the Act came into force I tried my best to persuade my committee that if the Act gave 'adequate medical relief without the taint of charity,' it would not be part of our job to spend subscribers' money in freely treating people for whom employers had compulsorily paid as insured persons. I knew enough to be aware that the Act did not give adequate medical relief in a broad sense, and that persons would have to come to the hospitals for adequate medical treatment. The treatment a doctor can give under the Act is largely defined by the capacity of a poor man to take medical treatment in his house. The docker living in one room—probably an attic—cannot have adequate medical treatment at home for many ailments, however skilful his doctor may be. There are many special departments—*x*-ray treatment, radiography, Finsen light, radium and electrical treatment, bacteriological tests, etc.—which are part of adequate medical treatment, but which the Act does not give. I took the line, and tried to persuade my committee to adopt it, that if we gave this treatment we ought to get payment from somebody, the friendly societies or someone else."

He then proceeds to state that he has been bound to modify his ground because the Government have defined "what is adequate medical treatment" as they intend it to be under the Insurance Act. It does not include such things as *x*-ray treatment, but only the average treatment which the average medical man can give. In minor cases the London Hospital will say to the patient: "This is nothing serious, and you can go to your doctor who will attend to you;" but in serious accidents or grievous illness the hospital must intervene and admit such cases, because the insured persons cannot otherwise obtain the adequate treatment of which they stand in urgent need. So the policy which the London Hospital intends to pursue is to admit all such cases whether they happen to be insured persons or not.

### The Provincial and Scottish General Hospitals.

Inferentially we have already stated the important differences between the provincial, the Scottish hospitals, the Royal Victoria Hospital, Belfast, and the voluntary general hospitals in London. The former voluntary hospitals depend for at least one-half of their ordinary revenues upon the free offerings of the working classes and of the army of small givers, whose case and position we have fully explained earlier in the course of this article. We have ventured to indicate certain directions in which modifications in the present system of treating the contributions received from these classes might with justice be adopted, so that the utmost inducement may be offered to the present subscribers, and especially to the two great groups indicated, to continue and even to increase their present contributions.

It is perfectly certain, in view of the statements made by the Chancellor to the London hospitals' deputation on the 17th instant, that the business-like and most prudent course for the managers of these groups of hospitals to pursue will be to continue to take insured persons as heretofore for a period of, say, twelve months, to keep a careful record of every insured person admitted after January 15, 1913, with particulars of the actual cost entailed by the treatment in hospital of this class of patient. They must steadily keep in view the fact that the administrative details on which the present system of management has been pursued in the past may be expected to require modification and change. Nothing will add more to the popularity of these hospitals, or tend to promote and strengthen their financial security, than the resolution that an honest and continuous attempt shall be initiated to make the introduction of national insurance the occasion for classifying their patients, by instituting free beds for the reception of the necessitous poor, and *pro rata* beds for the reception of the men sent in from contributing works. In this way it should be possible to introduce a system whereby the gifts of the benevolent can be placed to the credit of a free fund to defray the *pro rata* cost of maintaining the free beds in which the really necessitous poor will be treated, for these necessitous and deserving persons must ever have the first claim upon the voluntary hospitals. The contributions raised through working men's committees by free levies for a hospital should also be credited to a separate account for the maintenance of the beds occupied by patients sent in by the contributing works. When these modifications have been successfully carried there will remain for consideration and decision, how far and to what extent it may be possible to introduce a system of pay beds for the reception of clerks and other members of society, who have long felt the need of such provision, and can and will, we are confident, if opportunity is afforded them, gladly make some payment, or at least spontaneous levies, for the support of the hospitals on the same lines as those of the great working men's organisations as they exist to-day.

### The New System at St. Bartholomew's Hospital.

We have received the following resolutions adopted by the governors with regard to persons presenting themselves at St. Bartholomew's Hospital for treatment after January 15, 1913:—

1. That all persons coming to the Hospital (except in cases of urgent illness) should be asked by a lay official of the Hospital whether they are insured.

2. That each insured person should be referred to a Medical Officer of the Hospital to decide whether his or her ailment be urgent or not.

3. That in the case of an insured person with an ailment or accident which can be treated by a general practitioner of ordinary competence, such insured person should be told by a Medical Officer of the Hospital that he or she must obtain treatment by a Medical Practitioner outside the Hospital.

4. That the treatment of the really necessitous poor remain as at present.

5. That notices be posted in prominent positions in the Hospital in the following or similar terms, viz.: (*a*) On and after January 15, 1913, all persons applying for treatment will be required to state whether they are insured persons or not; (*b*) Insured persons with small ailments will not be treated at the Hospital.

We are in complete agreement with the policy which the above resolutions embody. They seem to us to meet the case admirably of the London hospitals with medical schools, though we consider they would be strengthened if the governors were free to resolve to make these resolutions provisional only for the next twelve months, so as to give ample time for experience to show to what extent, if at all, modifications may be necessary or desirable.

### A Practical Policy.

We are glad to note that the Council of the British Hospitals Association are meeting to-day (28th inst.) at St. Bartholomew's Hospital, West Smithfield, London, to receive a report of the Executive Committee in reference to the treatment of insured persons after January 15, 1913. That meeting will have the advantage of having before it important returns from the voluntary hospitals all over the country, relating to insured persons which will show the proportion of the patients at present under treatment at these institutions which come within this category. We observe the *Times* states that at the meeting of the representative body of the British Medical Association held on the 21st instant the scheme adopted by St. Bartholomew's Hospital (see above) was approved, and the Council was requested to make arrangements to obtain the approval of this scheme by all hospital staffs and committees throughout the country. It is of the first importance that the voluntary hospitals, so far as their circumstances will permit, should pursue a similar policy in regard to their attitude towards insured persons. We do not think it possible, for the reasons we have ventured to put forward in this article, that the voluntary hospitals outside London would be justified in binding themselves, at the moment, to adopt for all time the St. Bartholomew's Hospital scheme. We do feel, however, and are most desirous, as every thoughtful person must be, that everything should be done at this crisis to make it as easy as possible for insured persons to obtain consultative advice and special medical and surgical treatment at the voluntary hospitals; that the provincial and Scottish hospitals may properly agree to follow the lines of the St. Bartholomew's scheme as closely as the special circumstances of each hospital may allow, providing it is for a defined period only, say, twelve months, from the date when the benefits of the Insurance Act commence. This will allow opportunity for all the difficulties and special circumstances to be ascertained. That class of insured persons who, like the artisans, have by their spontaneous levies become entitled to hospital benefit, not as a matter of charity but of right, and the modifications which business considerations may make it imperative for hospital managers to introduce, whereby the wishes of the working classes in regard to *pro rata* payments for advice, treatment, and accommodation may be met, can be wisely considered meanwhile on their merits in all their bearings. Such a course should evolve in due course a practical policy of the first importance, whilst it should safeguard completely all the interests concerned.

